# Supplementary material for: COPD-Related Mortality before and after Mass COVID-19 Vaccination in Northern Italy
Source: Vaccines (Basel). 2023 Aug 21;11(8):1392. doi: 10.3390/vaccines11081392 (PMC10459975; doi:10.3390/vaccines11081392)

Table S1. Comorbidities* and place of death in COPD-related mortality: 2018-2019 average, 2020 and 2021.

	Average 2018-19	2020	2021
All COPD-related deaths, n	3,055	3,478	3,133
Comorbidities			
Diabetes	15%	17%	17%
Neoplasms	22%	21%	22%
Ischemic heart diseases	23%	21%	23%
Cerebrovascular diseases	11%	11%	10%
Hypertensive diseases	27%	27%	29%
Dementia/Alzheimer's disease	10%	10%	9%
Place of death			
Home	25%	27%	28%
Hospital	53%	52%	53%
Nursing home	17%	17%	15%

* ICD-10 codes: diabetes (E10-E14), neoplasms (C00-D48), ischemic heart diseases (I20-I25), cerebrovascular diseases (I60-I69), hypertensive diseases (I10-I13), dementia/Alzheimer's disease (F01-F03, G30)

Figure S1. Trend in the number of COPD-related deaths with mention of specific comorbidities during the pandemic.

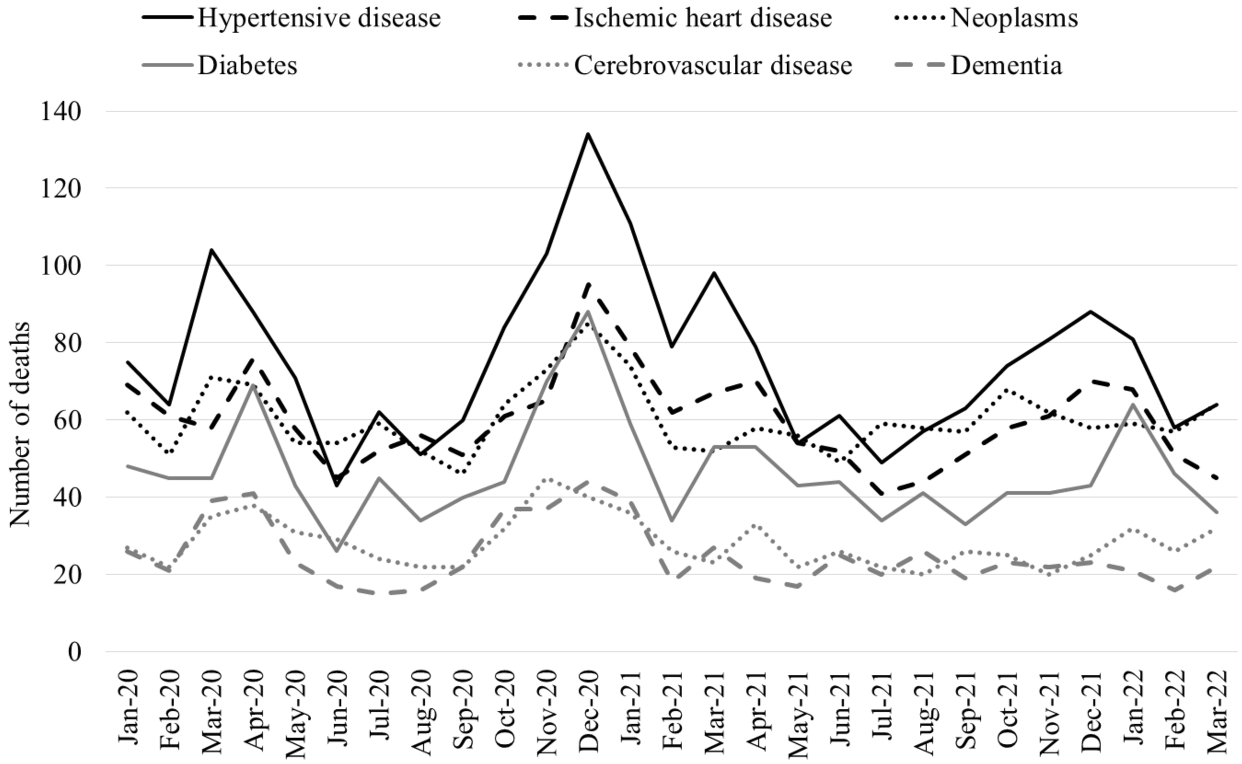

Figure S2. Monthly number of COPD-related deaths during the pandemic, by place of death.

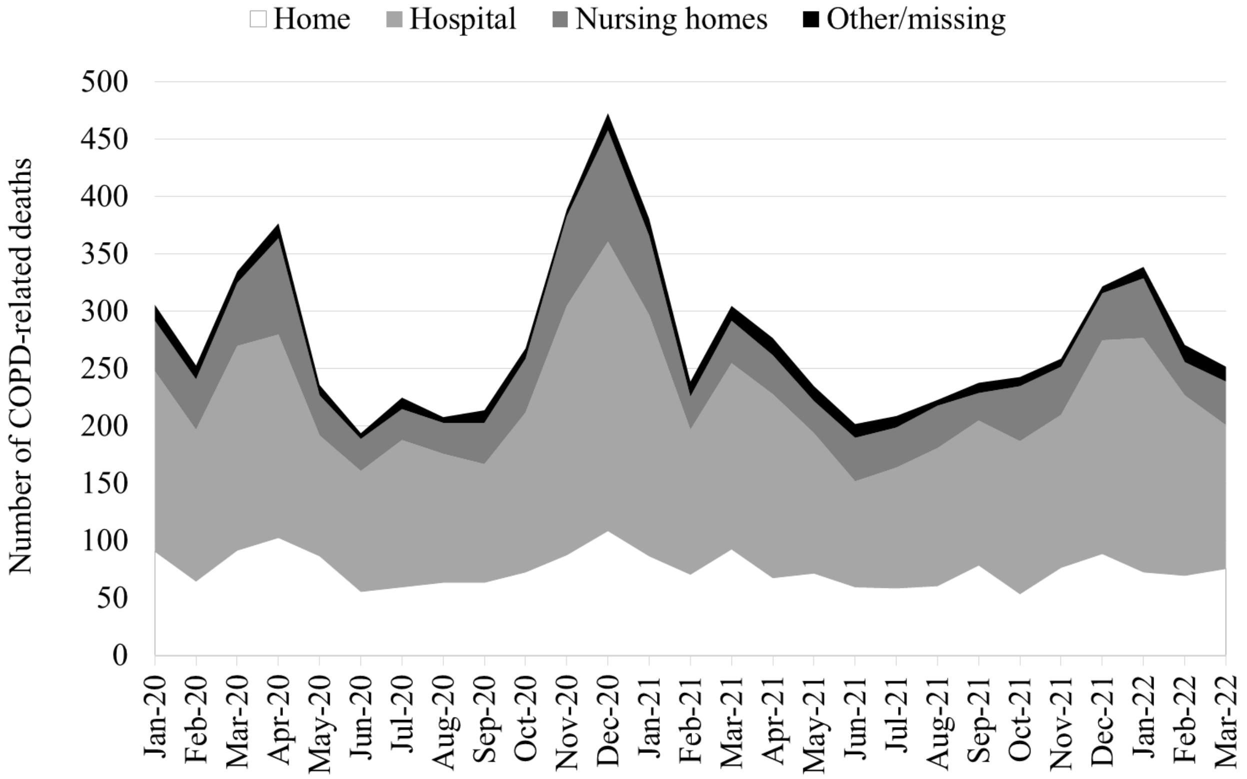

Supplement: Supplementary file 1 [file vaccines-11-01392-s001.zip › vaccines-2529214-supplementary.pdf]
